# Supplementary material for: A public antibody class recognizes an S2 epitope exposed on open conformations of SARS-CoV-2 spike
Source: Nat Commun. 2022 Aug 4;13:4539. doi: 10.1038/s41467-022-32232-0 (PMC9352689; doi:10.1038/s41467-022-32232-0)
Supplement: Supplementary file 1 — Supplementary Information [file 41467_2022_32232_MOESM1_ESM.pdf]

Figure S1

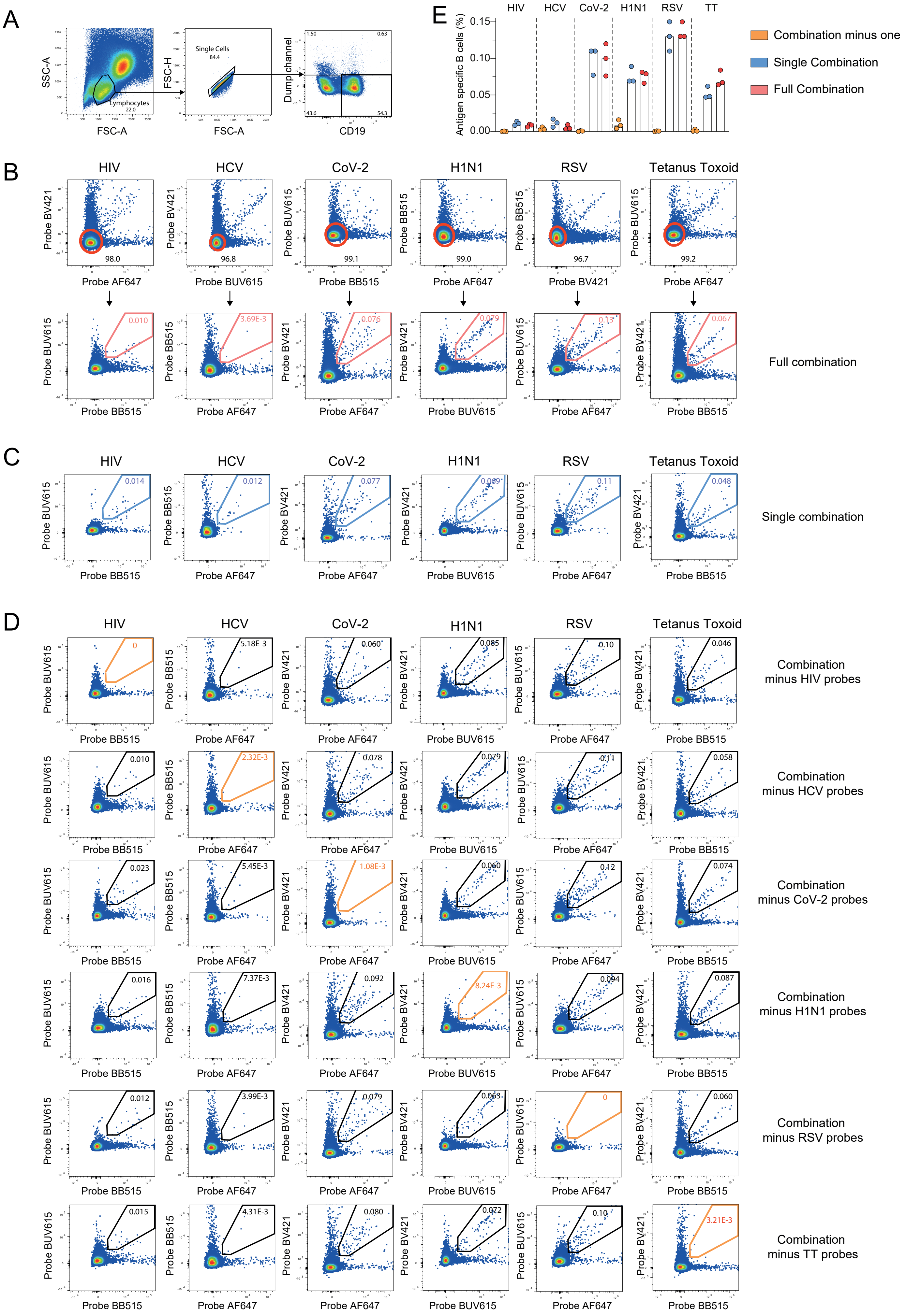
A
SSC-A
FSC-A
Lymphocytes
22.0
FSC-H
Single Cells
84.4
Dump channel
CD19
1.50
0.63
43.6
54.3
E
Antigen specific B cells (%)
HIV
HCV
CoV-2
H1N1
RSV
TT
Combination minus one
Single Combination
Full Combination
B
HIV
HCV
CoV-2
H1N1
RSV
Tetanus Toxoid
98.0
96.8
99.1
99.0
96.7
99.2
0.010
3.69E-3
0.076
0.079
0.13
0.067
Full combination
C
0.014
0.012
0.077
0.069
0.11
0.048
Single combination
D
Combination minus HIV probes
0
5.18E-3
0.060
0.085
0.10
0.046
Combination minus HCV probes
0.010
2.32E-3
0.078
0.079
0.11
0.058
Combination minus CoV-2 probes
0.023
5.45E-3
1.08E-3
0.060
0.12
0.074
Combination minus H1N1 probes
0.016
7.37E-3
0.092
8.24E-3
0.094
0.087
Combination minus RSV probes
0.012
3.99E-3
0.079
0.063
0
0.060
Combination minus TT probes
0.015
4.31E-3
0.080
0.072
0.10
3.21E-3
Probe BV421
Probe BUV615
Probe BB515
Probe AF647

Figure S1. Combinatorial probe staining strategy validation. (A) Gating strategy to identify live B cells. (B) Combinatorial probe staining and gating strategy for the detection of multiple B cell specificities in a single PBMC sample (see method section). Top panel: Each dot represents a live B cell. To remove potential cross-reactive B cells to streptavidin, each probe combination was first gated on cells double negative for the two other channels. Bottom panel: Antigen-specific B cells are then detected as double positive for the binding of the same antigen multimerized with two different fluorochromes according to a matrix code (Fig. 1A), all combinations were performed in a single sample (full combination). (C) Single combination: each single combination of antigen multimerized with two different fluorochromes was performed in independent samples. (D) Combination minus one: All combinations of antigen multimerized with two different fluorochromes minus one were performed in a single sample, removal of each single combination was tested to examine the robustness of the method. (E) Comparative analysis of antigen-specific frequencies within full combination, single combination and combination minus one. Each dot represents one individual experiment. Full combination conditions are similar to single combinations, and limited background is detected when a given combination is not added.

Figure S2

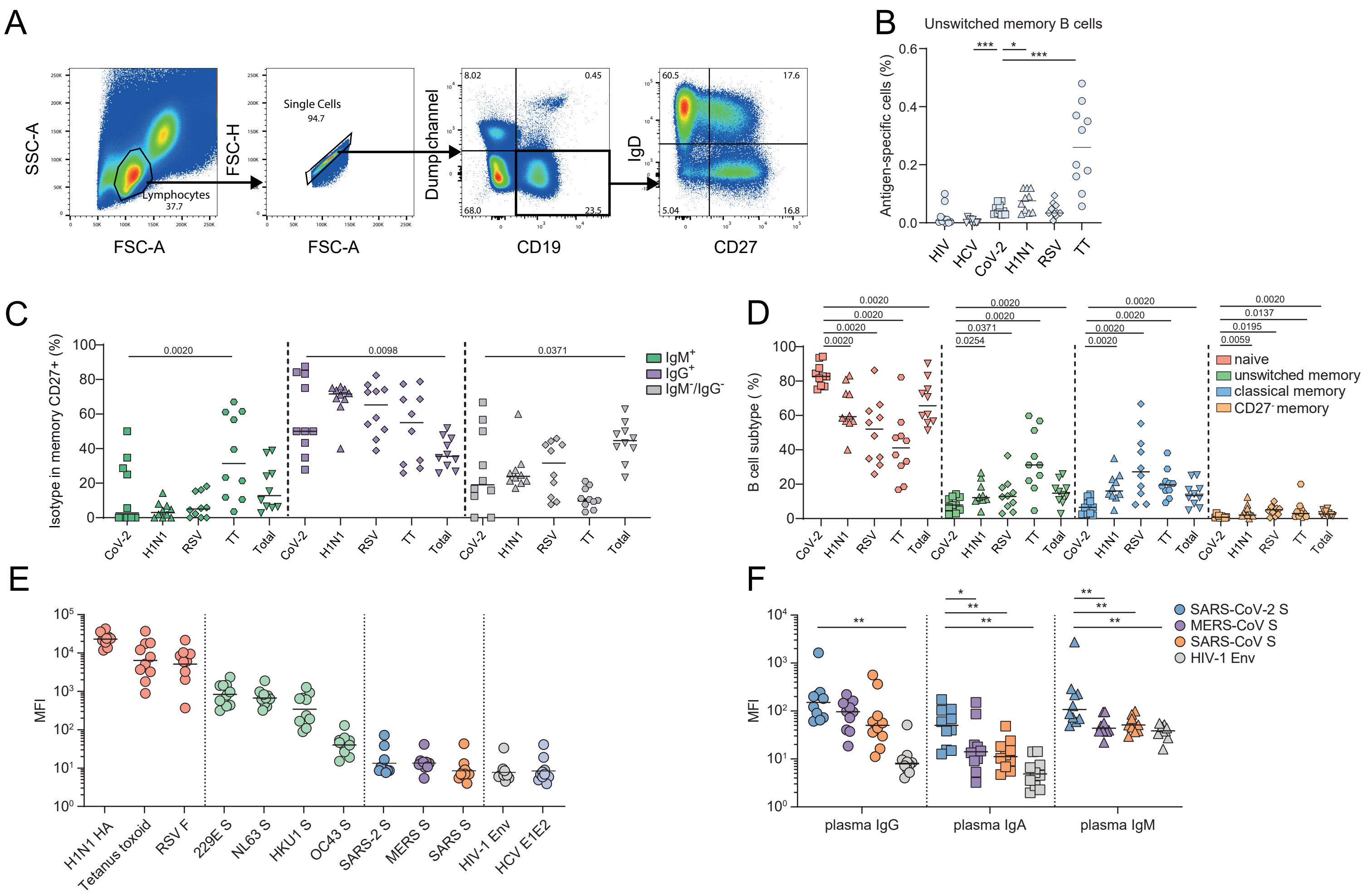

A
SSC-A
FSC-A
Lymphocytes
37.7
FSC-H
Single Cells
94.7
Dump channel
CD19
8.02
0.45
68.0
23.5
IgD
CD27
60.5
17.6
5.04
16.8
B
Unswitched memory B cells
Antigen-specific cells (%)
***
*
***
HIV
HCV
CoV-2
H1N1
RSV
TT
C
Isotype in memory CD27+ (%)
0.0020
0.0098
0.0371
IgM+
IgG+
IgM-/IgG-
CoV-2
H1N1
RSV
TT
Total
D
B cell subtype (%)
0.0020
0.0137
0.0195
0.0059
0.0371
0.0254
naive
unswitched memory
classical memory
CD27- memory
E
MFI
H1N1 HA
Tetanus toxoid
RSV F
229E S
NL63 S
HKU1 S
OC43 S
SARS-2 S
MERS S
SARS S
HIV-1 Env
HCV E1E2
F
MFI
**
*
plasma IgG
plasma IgA
plasma IgM
SARS-CoV-2 S
MERS-CoV S
SARS-CoV S
HIV-1 Env

Figure S2. Phenotypic characterization of SARS-CoV-2 S-reactive B cells in unexposed individuals extra data. (A) Gating strategies to identify live B cells (CD19+ Via- CD14-) and define B cell subsets according IgD and CD27 expression in total B cells (IgD+/CD27-, naive; IgD+/CD27+, unswitched IgD+ memory; IgD-/CD27-, CD27- memory; IgD-/CD27+, classical memory). (B-D) Statistical differences were tested only in comparison to SARS-CoV-2 condition (B) Analysis of frequency of antigen-specific B cells in the unswitched memory B cells population. (C) Analysis of frequency of IgG+, IgM+, or IgM-/IgG- in antigen-specific or Total classical memory B cells. (D) Analysis of frequency of antigen-specific B cells in the different B cell subsets (naive, unswitched memory, CD27- memory, classical memory). (E) MFI of plasma IgG binding (1:10.000 dilution) for each antigen as measured by custom Luminex assay. (F) MFI of plasma IgG, IgA and IgM binding (1:10 dilution) for each antigen as measured by custom Luminex assay. All statistical tests were performed with a Wilcoxon signed rank test for paired samples; *, $p < 0.05$; **, $p < 0.01$; ***, $p < 0.001$; ****, $p < 0.0001$.

Figure S3

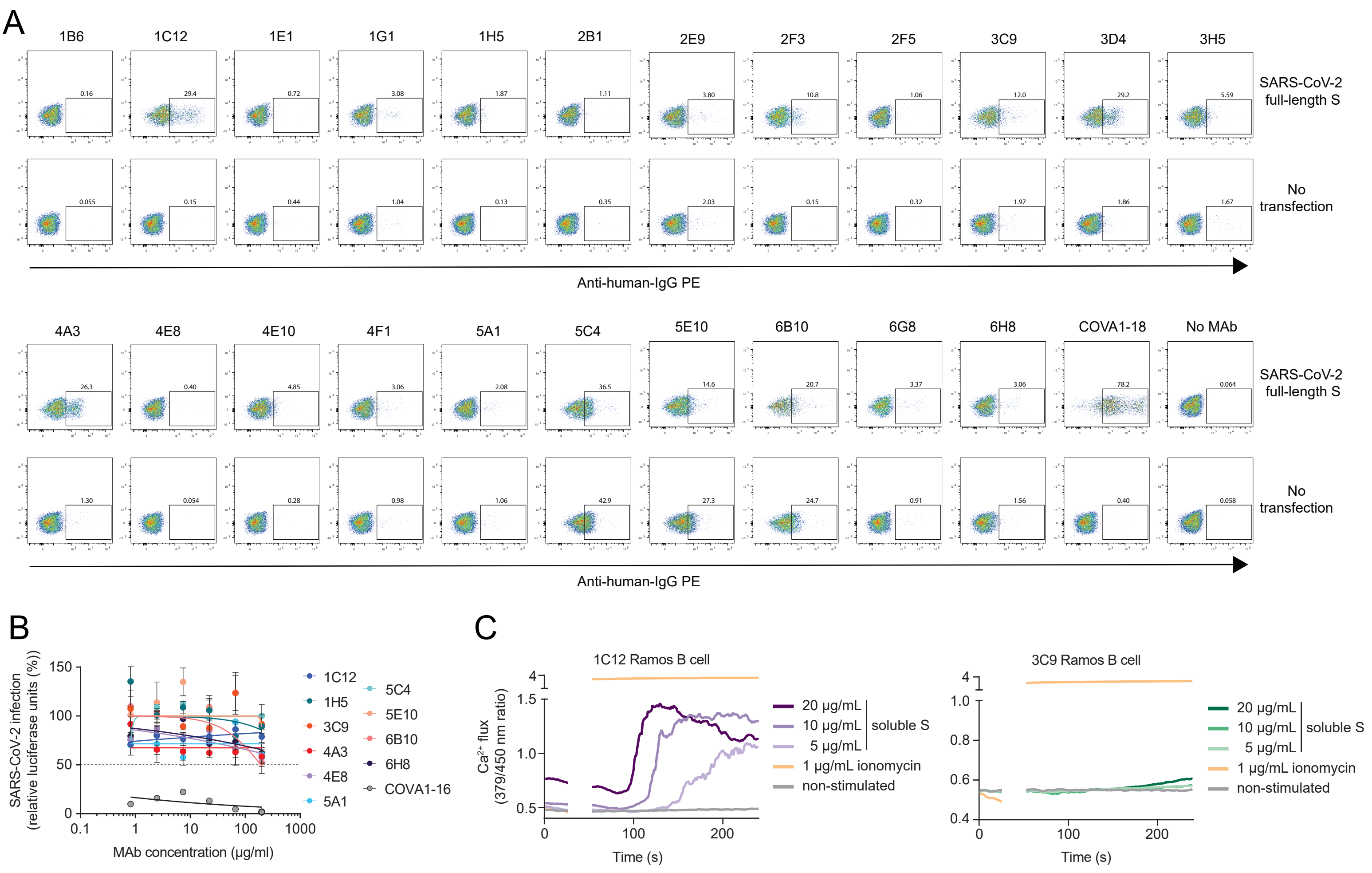
A
1B6
1C12
1E1
1G1
1H5
2B1
2E9
2F3
2F5
3C9
3D4
3H5
SARS-CoV-2 full-length S
No transfection
Anti-human-IgG PE
4A3
4E8
4E10
4F1
5A1
5C4
5E10
6B10
6G8
6H8
COVA1-18
No MAb
B
SARS-CoV-2 infection (relative luciferase units (%))
MAb concentration (µg/ml)
1C12
1H5
3C9
4A3
4E8
5A1
5C4
5E10
6B10
6H8
COVA1-16
C
1C12 Ramos B cell
3C9 Ramos B cell
Ca2+ flux (379/450 nm ratio)
Time (s)
20 µg/mL
10 µg/mL
5 µg/mL
soluble S
1 µg/mL ionomycin
non-stimulated

Figure S3. Molecular characterization of MAbs isolated from HDs. (A) FACS plots depicting binding to full length SARS-CoV-2 S-transfected or untransfected HEK293T cells for 23 selected MAbs isolated from unexposed individuals and control MAb COVA1-18 isolated from a convalescent COVID-19 patient. (B) Representative neutralization of SARS-CoV-2 pseudovirus of 10 selected MAbs isolated from unexposed individuals and control MAb COVA1-16 isolated from a convalescent COVID-19 patient. Error bars represent the standard deviation of technical replicates (n=3). (C) Ramos B cell activation of 1C12 B cells (top panel) and 3C9 B cells (bottom panel) as measured by calcium (Ca^{2+}) flux assay. A baseline without antigen was established between 0 and 30 seconds, after which the measurement was interrupted to add the antigen to the B cells (30-50 seconds). Ionomycin was used at 1 μg/mL as positive control.

Figure S4

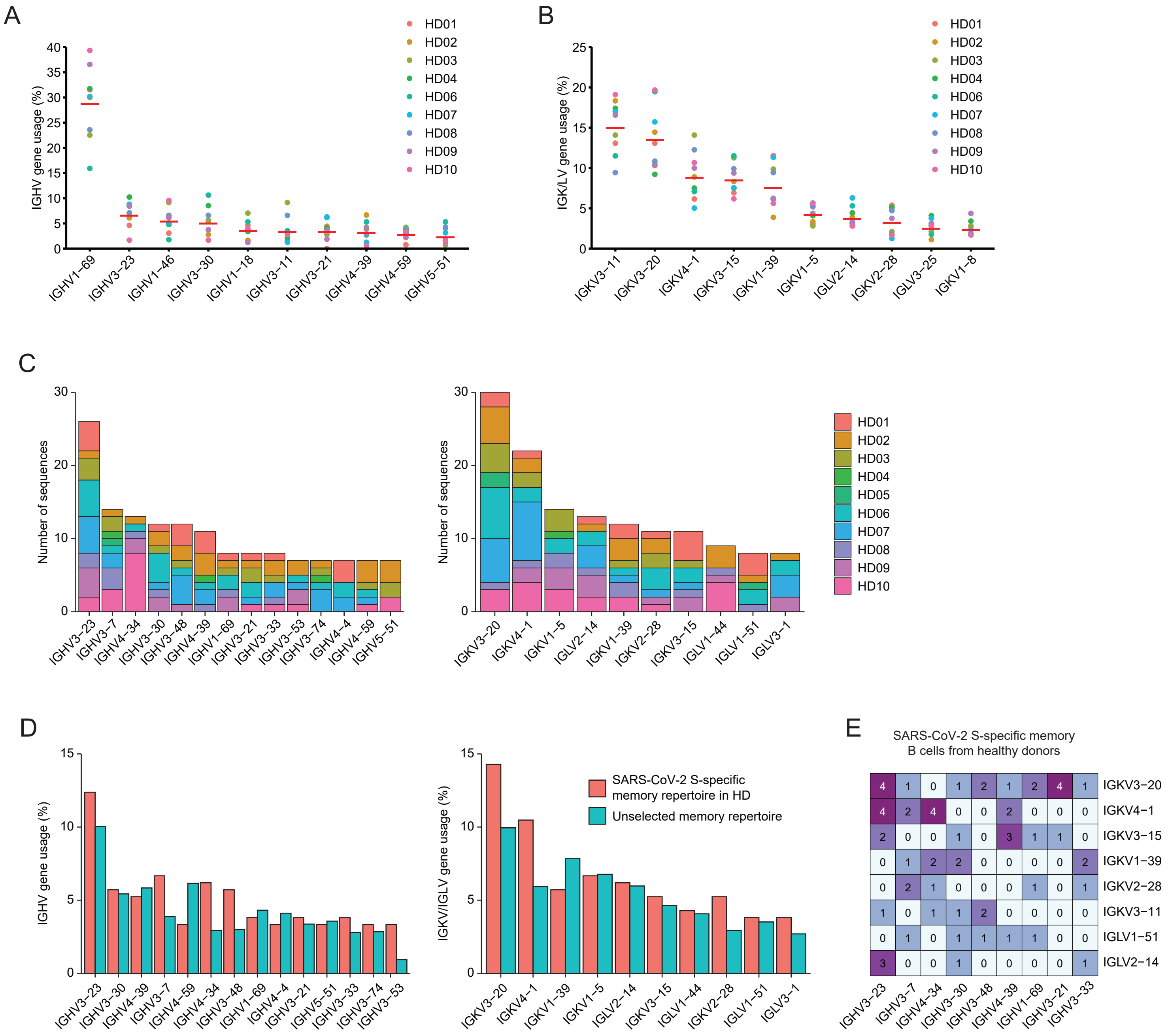
A
IGHV gene usage (%)
IGHV1-69
IGHV3-23
IGHV1-46
IGHV3-30
IGHV1-18
IGHV3-11
IGHV3-21
IGHV4-39
IGHV4-59
IGHV5-51
HD01
HD02
HD03
HD04
HD06
HD07
HD08
HD09
HD10
B
IGK/LV gene usage (%)
IGKV3-11
IGKV3-20
IGKV4-1
IGKV3-15
IGKV1-39
IGKV1-5
IGLV2-14
IGKV2-28
IGLV3-25
IGKV1-8
C
Number of sequences
IGHV3-23
IGHV3-7
IGHV4-34
IGHV3-30
IGHV3-48
IGHV4-39
IGHV1-69
IGHV3-21
IGHV3-33
IGHV3-53
IGHV3-74
IGHV4-4
IGHV4-59
IGHV5-51
IGKV3-20
IGKV4-1
IGKV1-5
IGLV2-14
IGKV1-39
IGKV2-28
IGKV3-15
IGLV1-44
IGLV1-51
IGLV3-1
HD01
HD02
HD03
HD04
HD05
HD06
HD07
HD08
HD09
HD10
D
IGHV gene usage (%)
IGKV/IGLV gene usage (%)
SARS-CoV-2 S-specific memory repertoire in HD
Unselected memory repertoire
IGHV3-23
IGHV3-30
IGHV4-39
IGHV3-7
IGHV4-59
IGHV4-34
IGHV3-48
IGHV1-69
IGHV4-4
IGHV3-21
IGHV5-51
IGHV3-33
IGHV3-74
IGHV3-53
IGKV3-20
IGKV4-1
IGKV1-39
IGKV1-5
IGLV2-14
IGKV3-15
IGLV1-44
IGKV2-28
IGLV1-51
IGLV3-1
E
SARS-CoV-2 S-specific memory B cells from healthy donors
4 1 0 1 2 1 2 4 1 IGKV3-20
4 2 4 0 0 2 0 0 0 IGKV4-1
2 0 0 1 0 3 1 1 0 IGKV3-15
0 1 2 2 0 0 0 0 2 IGKV1-39
0 2 1 0 0 0 1 0 1 IGKV2-28
1 0 1 1 2 0 0 0 0 IGKV3-11
0 1 0 1 1 1 1 0 0 IGLV1-51
3 0 0 1 0 0 0 0 1 IGLV2-14
IGHV3-23
IGHV3-7
IGHV4-34
IGHV3-30
IGHV3-48
IGHV4-39
IGHV1-69
IGHV3-21
IGHV3-33

Figure S4. BCR sequence analysis of memory B cells from HDs. (A-B) Depiction of IGHV (A) and IGK/LV (B) gene usage as in Fig. 3D-E, but for each individual donor as indicated in different colors. (C) Number of sequences in the memory cluster as defined in Fig. 3A for each respective IGHV and IGKV/IGLV gene. Each color corresponds to an individual HD. (D) IGHV (left panel) and IGKV/IGLV (right panel) gene usage in all recovered sequences in the memory cluster as defined in Fig. 3A (red) and an unselected memory repertoire from DeKosky et al. (35) (E) Matrix showing the number of pairs with a certain IGHV (x-axis) and IGKV (y-axis) recovered from SARS-CoV-2 S-reactive memory B cells from HDs.

Figure S5

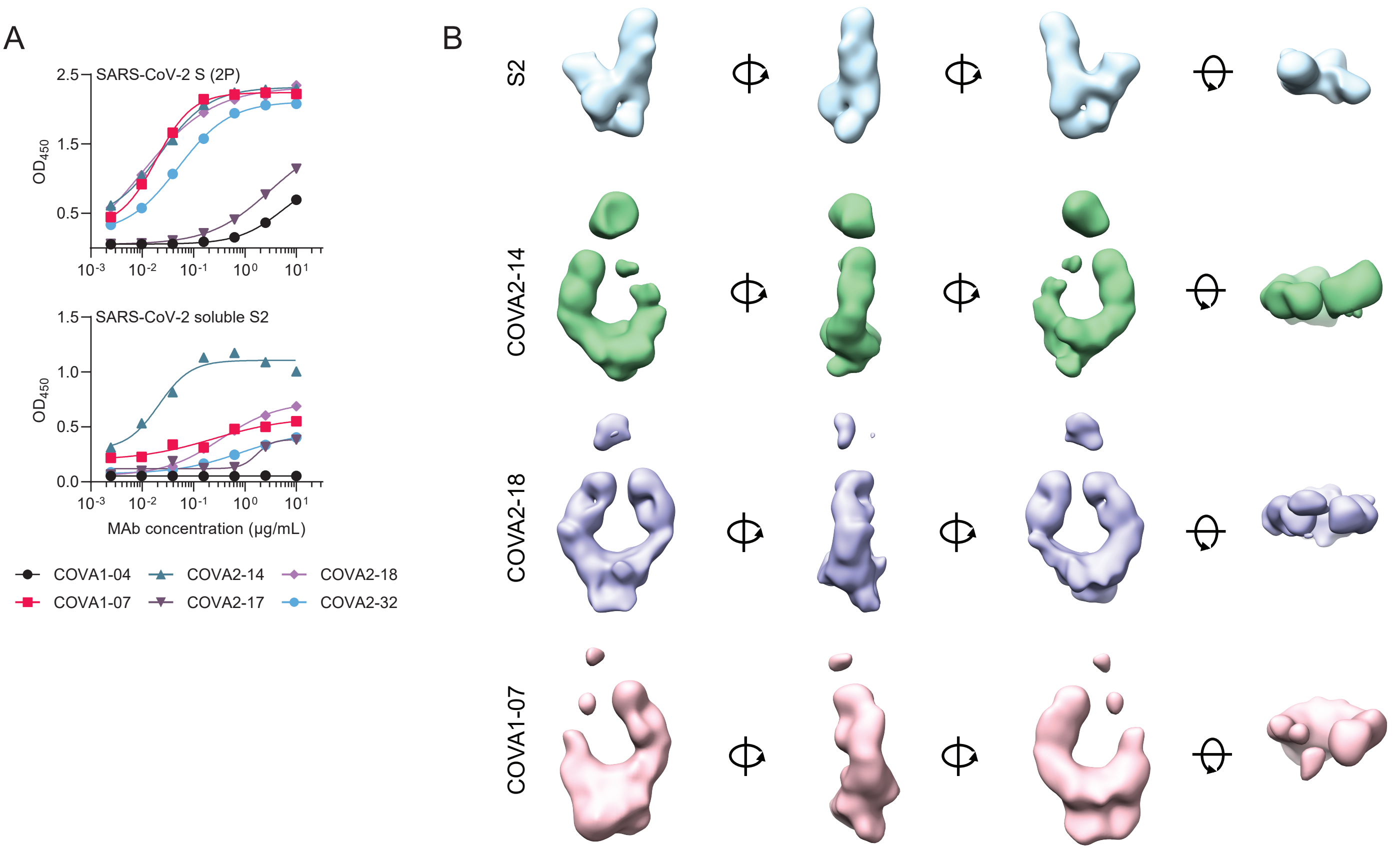
A
B
SARS-CoV-2 S (2P)
OD450
2.5
1.5
0.5
SARS-CoV-2 soluble S2
1.5
1.0
0.5
0.0
10-3
10-2
10-1
100
101
MAb concentration (μg/mL)
COVA1-04
COVA2-14
COVA2-18
COVA1-07
COVA2-17
COVA2-32
S2
COVA2-14
COVA2-18
COVA1-07

Figure S5. Characterization of IGHV1-69/IGKV3-11 MAbs from convalescent patients. (A) Enzyme-linked immunosorbent assay (ELISA) showing binding of six IGHV1-69/IGKV3-11 MAbs isolated from convalescent patients to SARS-CoV-2 S-2P (top panel) and soluble SARS-CoV-2 S2 (bottom panel). (B) 3D reconstruction SARS-CoV-2 S2 (top row) and COVA2-14, COVA2-18 and COVA1-07 MAbs in complex with SARS-CoV-2 S2.

Figure S6

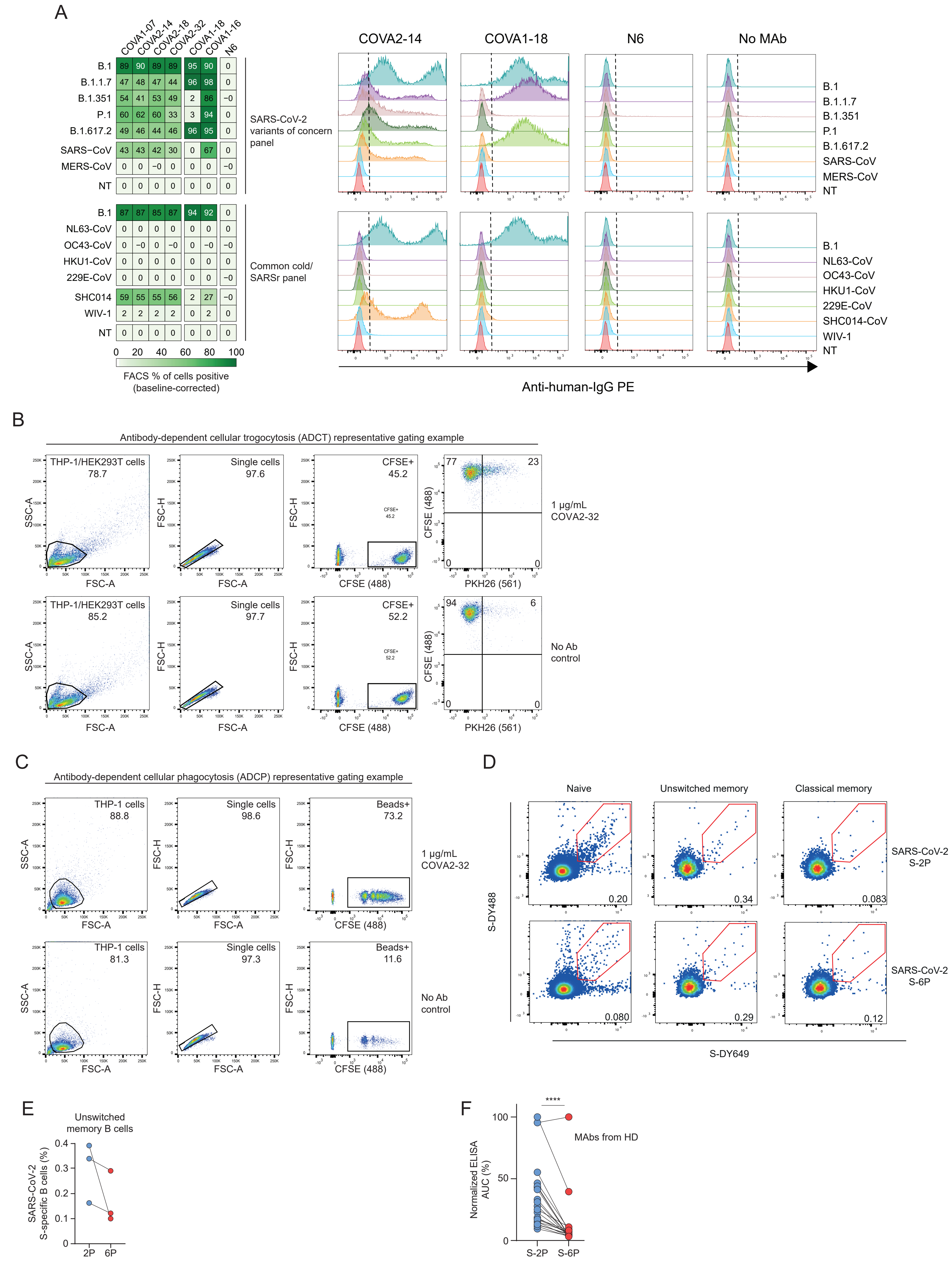
A
COVA1-07
COVA2-14
COVA2-18
COVA2-32
COVA1-18
COVA1-16
N6
B.1 89 90 89 89 95 90 0
B.1.1.7 47 48 47 44 96 98 0
B.1.351 54 41 53 49 2 86 −0
P.1 60 62 60 33 3 94 0
B.1.617.2 49 46 44 46 96 95 0
SARS−CoV 43 43 42 30 0 67 0
MERS-CoV 0 0 −0 0 0 0 −0
NT 0 0 0 0 0 0 0
SARS-CoV-2 variants of concern panel
B.1 87 87 85 87 94 92 0
NL63-CoV 0 0 0 0 0 0 0
OC43-CoV 0 −0 0 −0 0 −0 −0
HKU1-CoV 0 0 0 0 0 0 0
229E-CoV 0 0 0 0 0 0 −0
SHC014 59 55 55 56 2 27 −0
WIV-1 2 2 2 2 0 2 0
NT 0 0 0 0 0 0 0
Common cold/ SARSr panel
0 20 40 60 80 100
FACS % of cells positive (baseline-corrected)
COVA2-14
COVA1-18
N6
No MAb
B.1
B.1.1.7
B.1.351
P.1
B.1.617.2
SARS-CoV
MERS-CoV
NT
B.1
NL63-CoV
OC43-CoV
HKU1-CoV
229E-CoV
SHC014-CoV
WIV-1
NT
Anti-human-IgG PE
B
Antibody-dependent cellular trogocytosis (ADCT) representative gating example
THP-1/HEK293T cells 78.7
Single cells 97.6
CFSE+ 45.2
77
23
0
0
1 µg/mL COVA2-32
THP-1/HEK293T cells 85.2
Single cells 97.7
CFSE+ 52.2
94
6
0
0
No Ab control
SSC-A
FSC-H
FSC-A
CFSE (488)
PKH26 (561)
C
Antibody-dependent cellular phagocytosis (ADCP) representative gating example
THP-1 cells 88.8
Single cells 98.6
Beads+ 73.2
1 µg/mL COVA2-32
THP-1 cells 81.3
Single cells 97.3
Beads+ 11.6
No Ab control
SSC-A
FSC-H
FSC-A
CFSE (488)
D
Naive
Unswitched memory
Classical memory
0.20
0.34
0.083
SARS-CoV-2 S-2P
0.080
0.29
0.12
SARS-CoV-2 S-6P
S-DY488
S-DY649
E
Unswitched memory B cells
SARS-CoV-2 S-specific B cells (%)
0 0.1 0.2 0.3 0.4
2P
6P
F
****
MAbs from HD
Normalized ELISA AUC (%)
0 50 100
S-2P
S-6P

Figure S6. Functional characterization of IGHV1-69/IGKV3-11 MAbs. (A) Matrix showing flow cytometric binding assay to SARS-CoV-2 S-transfected or untransfected HEK293T cells (NT) for IGHV1-69/IGKV3-11 MAbs (COVA1-07, COVA2-14, COVA2-18 and COVA2-32), anti-SARS-CoV-2 MAbs isolated from convalescent patients (COVA1-18 and COVA1-16) and control anti-HIV-1 MAb N6. Numbers and colors in the boxes represent the percentage of cells showing binding to a particular MAb. Representative MFI peaks are shown for COVA2-14, COVA1-18, N6 and no MAb are shown on the right. (B-C) Representative gating strategy for antibody-dependent cellular trogocytosis (ADCT, (B)) and antibody-dependent cellular phagocytosis (ADCP, (C)). The top panels show an example of COVA2-32, the bottom panels show the no MAb controls. (D) Representative gating strategy showing binding of naive (left), unswitched memory (middle) and classical memory (right) B cells to SARS-CoV-2 S-2P in two colors (top row) and SARS-CoV-2 S-6P in two colors (bottom row). (E) Connected dot plots showing the frequency of unswitched B cells of SARS-CoV-2 S-2P- or S-6P-specific B cells (%) in three HDs. (F) Connected dot plot showing the ELISA area under the curve (AUC) of isolated MAbs from HDs to SARS-CoV-2 S-2P- or S-6P. Non-parametric Wilcoxon signed-rank test with ****, $p < 0.0001$.
