# Supplementary material for: A public antibody class recognizes an S2 epitope exposed on open conformations of SARS-CoV-2 spike
Source: Nat Commun. 2022 Aug 4;13:4539. doi: 10.1038/s41467-022-32232-0 (PMC9352689; doi:10.1038/s41467-022-32232-0)
Supplement: Supplementary file 3 — Description to Additional Supplementary Information [file 41467_2022_32232_MOESM3_ESM.pdf]

Description of Additional Supplementary Files

Supplementary Data 1: Monoclonal antibodies

Table depicting the properties of BCR sequences and subsequent monoclonal antibodies derived from single-cell sorted SARS-CoV-2 S-reactive B cells of four unexposed donors (HD01-HD04). Each row depicts heavy and light chain BCR sequences and subsequent produced monoclonal antibody properties from one single cell. Columns depict the unique identifiers for each cells; Gene usage of the variable, junction and diversity from heavy and light chains; CDRH3 and CDRL3 lengths and amino acid compositions; The B cell subtype the single cell originated from, Naïve, classical memory, unswitched memory B cells; The Monoclonal antibody binding properties to SARS-CoV-2 spike (qualitative: yes/No), as well as the binding (IC:50) to unrelated antigens (Insulin, LPS, Cardiolipin, Calf thymus dsDNA, Salmonella flagellin).
