# Supplementary material for: A public antibody class recognizes an S2 epitope exposed on open conformations of SARS-CoV-2 spike
Source: Nat Commun. 2022 Aug 4;13:4539. doi: 10.1038/s41467-022-32232-0 (PMC9352689; doi:10.1038/s41467-022-32232-0)
Supplement: Supplementary file 5 — Reporting Summary [file 41467_2022_32232_MOESM5_ESM.pdf]

nature portfolio

Corresponding author(s): Marit van Gils and Rogier Sanders

Last updated by author(s): Mar 9, 2022

Reporting Summary

Nature Portfolio wishes to improve the reproducibility of the work that we publish. This form provides structure for consistency and transparency in reporting. For further information on Nature Portfolio policies, see our Editorial Policies and the Editorial Policy Checklist.

Statistics

For all statistical analyses, confirm that the following items are present in the figure legend, table legend, main text, or Methods section.

n/a	Confirmed	
☐	☒	The exact sample size (n) for each experimental group/condition, given as a discrete number and unit of measurement
☐	☒	A statement on whether measurements were taken from distinct samples or whether the same sample was measured repeatedly
☐	☒	The statistical test(s) used AND whether they are one- or two-sided<br>*Only common tests should be described solely by name; describe more complex techniques in the Methods section.*
☒	☐	A description of all covariates tested
☐	☒	A description of any assumptions or corrections, such as tests of normality and adjustment for multiple comparisons
☐	☒	A full description of the statistical parameters including central tendency (e.g. means) or other basic estimates (e.g. regression coefficient) AND variation (e.g. standard deviation) or associated estimates of uncertainty (e.g. confidence intervals)
☐	☒	For null hypothesis testing, the test statistic (e.g. F, t, r) with confidence intervals, effect sizes, degrees of freedom and P value noted<br>*Give P values as exact values whenever suitable.*
☒	☐	For Bayesian analysis, information on the choice of priors and Markov chain Monte Carlo settings
☒	☐	For hierarchical and complex designs, identification of the appropriate level for tests and full reporting of outcomes
☒	☐	Estimates of effect sizes (e.g. Cohen's d, Pearson's r), indicating how they were calculated

Our web collection on statistics for biologists contains articles on many of the points above.

Software and code

Policy information about availability of computer code

Data collection	Flow cytometry data were aquired using LSR Fortessa III and FACS ARIA II using BD FACS DIVA software, Flowjo v10.7 software, was used to analyzed the data. Single cell RNAseq data was analyzed with the proprietary 10X Genomics platform.
Data analysis	Statistical analytsis was performed using Graphpad prism v9.0 software. 10X single cell RNAseq was analyzed with R version 4.0.3 and the full code can be found at a public repository https://github.com/caniels/hdpaper/.

For manuscripts utilizing custom algorithms or software that are central to the research but not yet described in published literature, software must be made available to editors and reviewers. We strongly encourage code deposition in a community repository (e.g. GitHub). See the Nature Portfolio guidelines for submitting code & software for further information.

Data

Policy information about availability of data

All manuscripts must include a data availability statement. This statement should provide the following information, where applicable:

- Accession codes, unique identifiers, or web links for publicly available datasets
- A description of any restrictions on data availability
- For clinical datasets or third party data, please ensure that the statement adheres to our policy

All data is readily available in the main text and supplementary materials. Supplementary information files (Source Data file) related to single cell RNAseq analysis will be available at NCBI GEO before publication under the accession number GSE196820. NS-EM reconstructions are deposited to the Electron Microscopy Data Bank under the accession number EMD26217-EMD26220. All reasonable requests materials used in this study should be directed to and will be fulfilled under an MTA by Prof. Rogier W Sanders (r.w.sanders@amsterdamumc.nl) and Dr. Marit J van Gils (m.j.vangils@amsterdamumc.nl).

Field-specific reporting

Please select the one below that is the best fit for your research. If you are not sure, read the appropriate sections before making your selection.

☒ Life sciences ☐ Behavioural & social sciences ☐ Ecological, evolutionary & environmental sciences

For a reference copy of the document with all sections, see nature.com/documents/nr-reporting-summary-flat.pdf

Life sciences study design

All studies must disclose on these points even when the disclosure is negative.

Sample size	The sample size was based on the of the avaibility of the samples: 10 pre-pandemic donors (unvaccinated/uninfected by SARS-CoV-2) were available at the time of study.
Data exclusions	Sequences from NGS data were quality checked and doublets were excluded.
Replication	Experiments on primary B cells were performed with 10 samples, other experiments were all confirmed 2-3 times and in some cases only representative experiments/replications are shown. All attempts at replication were successful.
Randomization	Randomization was not relevant for this study, as we did not perform individual group comparison and the research question was open-ended.
Blinding	Blinding was not relevant for this study, as we did not perform individual group comparison and the research question was open-ended.

Reporting for specific materials, systems and methods

We require information from authors about some types of materials, experimental systems and methods used in many studies. Here, indicate whether each material, system or method listed is relevant to your study. If you are not sure if a list item applies to your research, read the appropriate section before selecting a response.

Materials & experimental systems

n/a	Involved in the study	
☐	☒	Antibodies
☐	☒	Eukaryotic cell lines
☒	☐	Palaeontology and archaeology
☒	☐	Animals and other organisms
☐	☒	Human research participants
☒	☐	Clinical data
☒	☐	Dual use research of concern

Methods

n/a	Involved in the study	
☒	☐	ChIP-seq
☐	☒	Flow cytometry
☒	☐	MRI-based neuroimaging

Antibodies

Antibodies used	eBioscience™ Fixable Viability Dye eFluor™ 780 - clone N/A<br>CD4 Antibody, APC-eFluor™ 780 (#47-0048-42) - Thermo Fisher - clone OKT4<br>Alexa Fluor® 700 anti-human CD19 Antibody (#302225) - BioLegend - clone HIB19<br>Brilliant Violet 785™ anti-human IgD Antibody (#348241) - BioLegend - clone IA6-2<br>PE Mouse Anti-Human CD27 (#555441) - BD Biosciences - clone M-T271<br>PE-Cy™7 Mouse Anti-Human IgG (#561298) - BD Biosciences - clone G18-145<br>BUV395 Rat Anti-Mouse IgM (#564025) - BD Biosciences - clone G20-127<br>CD3 Antibody, APC-eFluor™ 780 (47-0038-42) - Thermo Fisher - clone UCHT1<br>CD14 Antibody, APC-eFluor™ 780 (47-0149-42) - Thermo Fisher - clone C1D3<br>CD16 Antibody, APC-eFluor™ 780 (47-0168-42) - Thermo Fisher - clone CB16<br>TotalSeq-C0251_260 anti-human Hashtag 1-10 Antibody (#394661-39470) - Biolegend - clone LNH-94; 2M2<br>TotalSeq™-C0384 anti-human IgD Antibody (#348245) - Biolegend - clone IA6-2<br>TotalSeq™-C0154 anti-human CD27 Antibody (#302853) - BioLegend - clone O323<br>Brilliant Violet 605™ anti-human IgM Antibody (#314524) - Biolegend - clone MHM-88
Validation	All primary antibodies for the application and species reactivity were validated by the manufacturer (see link to manufacturer websites below). Antibody were titrated for best ratio saturation/noise<br>https://www.thermofisher.com/order/catalog/product/65-0865-14<br>https://www.thermofisher.com/antibody/product/CD4-Antibody-clone-OKT4-OKT-4-Monoclonal/47-0048-42<br>https://www.biolegend.com/it-it/products/alexa-fluor-700-anti-human-cd19-antibody-3399<br>https://www.biolegend.com/it-it/products/brilliant-violet-785-anti-human-igd-antibody-13612

https://www.bdbiosciences.com/en-nl/products/reagents/flow-cytometry-reagents/research-reagents/single-color-antibodies-ruo/pe-mouse-anti-human-cd27.555441
https://www.bdbiosciences.com/en-nl/products/reagents/flow-cytometry-reagents/research-reagents/single-color-antibodies-ruo/pe-cy-7-mouse-anti-human-igg.561298
https://www.bdbiosciences.com/en-nl/products/reagents/flow-cytometry-reagents/research-reagents/single-color-antibodies-ruo/buv395-rat-anti-mouse-igm.564025
https://www.thermofisher.com/antibody/product/CD3-Antibody-clone-UCHT1-Monoclonal/47-0038-42
https://www.thermofisher.com/antibody/product/CD14-Antibody-clone-61D3-Monoclonal/47-0149-42
https://www.thermofisher.com/antibody/product/CD16-Antibody-clone-eBioCB16-CB16-Monoclonal/47-0168-42
https://www.biolegend.com/fr-ch/products/totalseq-c0251-anti-human-hashtag-1-antibody-17162
https://www.biolegend.com/it-it/products/totalseq-c0384-anti-human-igd-antibody-17865
https://www.biolegend.com/it-it/products/totalseq-c0154-anti-human-cd27-antibody-16840
https://www.biolegend.com/it-it/products/brilliant-violet-605-anti-human-igm-antibody-8746

Eukaryotic cell lines

Policy information about cell lines

Cell line source(s)	Drs. Li Wu and Vineet N. Kewal Ramani from the NIH AIDS Reagent Program provided Ramos B cells. HEK293T cells and HEK293F cells were provided by ATTC and Invitrogen respectively.
Authentication	Ramos cell lines, HEK293T, and HEK293F were authenticated by the provider (ThermoFisher) and Ramos cells phenotype was validated by FACS.
Mycoplasma contamination	All cell lines tested negative for mycoplasma.
Commonly misidentified lines (See ICLAC register)	No commonly misidentified cell lines were used in the study.

Human research participants

Policy information about studies involving human research participants

Population characteristics	10 anonymous healthy donor samples from the Dutch national blood bank Sanquin. No information was disclosed on these individuals.
Recruitment	No specific and/or targeted recruitment is used in this study.
Ethics oversight	Patient consent was waived due to anonymized donation of blood for blood donation, blood products and research by the donors to the Dutch national blood bank Sanquin. No ethics board was involved as this is not required when donating blood and/or blood products at Sanquin.

Note that full information on the approval of the study protocol must also be provided in the manuscript.

Flow Cytometry

Plots

Confirm that:

- [x] The axis labels state the marker and fluorochrome used (e.g. CD4-FITC).
- [x] The axis scales are clearly visible. Include numbers along axes only for bottom left plot of group (a 'group' is an analysis of identical markers).
- [x] All plots are contour plots with outliers or pseudocolor plots.
- [x] A numerical value for number of cells or percentage (with statistics) is provided.

Methodology

Sample preparation

Antigen-specific detection acquisition and sorting:

General protocol:
Frozen PBMC samples were first depleted for T cells using CD3 selection kit II (StemCell) according to the manufacturer's instruction. Enriched B cells or Ramos B cell lines were then stained in Eppendorf tubes with 50-100 µL of antigen probe cocktail for 30 min at 4°C, subsequently washed with FACS buffer (PBS supplemented with 1 mM EDTA and 2% fetal calf) and stained with the Live/DEAD dye together with MAbs coupled with fluorophores for FACS (Table S1) for an additional 30 min at 4°C. Stained samples were washed twice and acquired on FACS

B cell sorting for 10xgenomics
Enriched B cells from 4×107 PBMCs of 10 donor were stained with Human TruStain FcX Fc Blocking Reagent (BioLegend, 422302) for 10 min at 4°C. For each donor a mix of Abs linked to feature barcodes, containing one specific hashtag barcode

together with CD27 and IgD, was centrifuged at 14,000g at 4°C for 10min and supernatant harvested. Barcoded antibody mix, anti-CD19-AF700, Live/DEAD dye, and labelled SARS-CoV-2 S (AF647 and BV421) were added to the cells and stained for 30min at 4°C. Cells were then washed twice and resuspended in FACS buffer before facs acquisition

B cell activation experiments of Ramos B cells
4×106 cells/mL in RPMI10 were loaded with 1.5 µM of the calcium indicator Indo-1 (Invitrogen) for 30 min at 37°C, washed with Hank's Balance Salt Solution supplemented with 2 mM CaCl2, followed by another incubation of 30 min at 37°C. Antigen-induced Ca2+ influx of B cells was monitored on a LSR Fortessa by measuring the 379/450 nm emission ratio of Indo-1 fluorescence upon UV excitation. Following 30 s of baseline measurement, aliquots of 1×106 cells/mL were then stimulated for 210 s at RT with either 20 µg/mL, 10 µg/mL or 5 µg/mL of SARS-CoV-2 S or the equimolar amount presented on I53-50NPs. Ionomycin (Invitrogen) was added to a final concentration of 1 µg/µL to determine the maximum Indo-1-fluorescence.

Binding assays to cell surface expressed CoV-S :
HEK293T cells were transfected with full length S plasmid DNA (SARS-CoV-2 WT and variants, other epidemic and endemic CoVs, and Bat-CoVs) using Lipofectamine2000 (Invitrogen). Briefly, 0.5×106 cells/well were plated in a 6-well plate. After 24h, 4 µg DNA and 10 µL lipofectamine were mixed, incubated and added to each well. After 48 h, cells were harvested and pooled, and 5×104 cells were incubated in RPMI with 50 µg/mL of purified SARS-CoV-2 S-reactive MAbs for 1 h at RT. Cells were subsequently washed twice with PBS and stained for 30 min on ice and in the dark, in 50 µL of FACS buffer containing 1:1000 diluted PE- conjugated goat anti-human IgG (Biolegend). Cells were then washed twice with FACS buffer, fixed with 2% PFA and subsequently analyzed on BD LSRFortessa.

Antibody-dependent cellular trogocytosis:
HEK293F cells (Invitrogen) at a density of 1×106cells/mL were transfected using SARS-CoV-2 S plasmid and PEImax (1 µg/µl) in a 3:1 ratio in OptiMEM. HEK293F cells were harvested 72 hours after transfection and their plasma membrane was stained with 10µM PKH26 (Sigma-Aldrich) dye in PBS, for 20 min (RT) with periodic mixing. Cells were washed twice with PBS and taken up in culture medium. THP-1 effector cells (ATCC) were stained intracellularly with 0.05µM carboxyfluorescein succinimidyl ester (CFSE, ThermoFisher) in PBS and incubate 20 min (RT) with periodic mixing. Cells were washed twice with PBS and taken up in culture medium.). PKH26 stained HEK293F cells were opsonized for 30 min at 37°C, with serial MAb dilutions. 2G12-IgG1, specific for HIV-1 gp120, was used as a negative control. After incubation, cells were washed and THP-1 cells were added to the HEK293F cells at a 2:1 effector:target ratio. Plates were centrifuged shortly to promote cell to cell contact and incubated 1 hour at 37°C. Afterwards, cells were washed and resuspended in PBS/2% FCS and aquired on FACS.

Antibody-dependant cellular phagocytosis
Fluorescent neutravidin beads (Invitrogen) were incubated with biotinylated SARS-CoV-2 S-2P or RBD protein overnight at 4 °C. Beads were subsequently centrifuged shortly and washed twice with PBS/2% BSA to remove unbound antigen and block the remaining hydrophobic sites on the microspheres. The coated beads were resuspended in PBS/2% BSA and 0.1 µL of the original suspension was placed in every well of a V-bottom 96 well plate and incubated (2 h at 37°C) with serial MAb dilutions. 2G12-IgG1, specific for HIV-1 gp120, was used as a negative control. After incubation, plates were washed and 5×104THP-1 effector cells (ATCC) were added to each well in a final volume of 100 µL of RPMI10. Subsequently, plates were centrifuged shortly to promote beads to cell contact before incubation (5 h at 37°C). After incubation, the cells were washed, resuspended in PBS/2% FCS and analyzed by flow cytometry.

Instrument

Flow cytometry data were aquired using LSR Fortessa III and FACS ARIA II

Software

BD FACS DIVA software, Flowjo v10.7 software

Cell population abundance

Purity of magnetically enriched B cells was confirmed by flow cytometry. Purity of sorted antigen-specific B cells for MAbs isolation and single cell RNA-seq could not be assessed due to the scarcity of the population, no post-sort acquisition was performed, all sorted samples were used for further analysis.

Gating strategy

Combinatorial probe gating strategy:
Conventionally, antigen-specific B cells are detected by the binding of two different fluorochrome-coded to the same protein. In our study, we were able to detect 6 different antigen-specificities using 4 distinct fluorophores. By using a combinatorial probe staining strategy: SARS-CoV-2 S (AF647, BV421), H1N1 HA (BUV615, BV421), RSV F (AF647, BUV615), HCV E1E2 (AF647, BB515), HIV-1 ConM Env (BB515, BUV615) and Tetanus toxoid (BB515, BV421). For the analysis, the lymphocyte population was first gated based on the morphology (FSC-A/SSC-A) and doublets were removed. Next, dead cells and remaining CD4+ cells to avoid artefact binding of HIV-1 probes were first excluded within a dump channel and live antigen-specific B cells were studied in the CD19+population. To remove potential cross-reactive B cells to streptavidin, each probe combination was first gated on cells double negative for the two other channels (Fig. S1).

B cell sorting for MAb isolation
The lymphocyte population was first gated based on the morphology (FSC-A/SSC-A) and doublets were removed. Dead cells and non-B cells were first excluded within a dump channel (CD3-/CD14-/CD16-). Live B cells (CD19+) that were double positive for the SARS-CoV-2 S protein (AF647 and BV421) were single cell-sorted. Using index sorting, sorted cells were evaluated for their expression of IgD and CD27 and characterized as naive B cells (IgD+ CD27-), Double negative (IgD- CD27-), Classical memory (IgD- CD27+) or Unswitched memory B cells (IgD+ CD27+)

B cell sorting for 10xgenomics
The lymphocyte population was first gated based on the morphology (FSC-A/SSC-A) and doublets were removed. Dead cells were first excluded. Live B cells positives for the SARS-CoV-2 S protein (AF647 and BV421) were bulk sorted

Ramos B cell lines:
Ramos cells were first gated based on the morphology (FSC-A/SSC-A) and doublets were removed. Live cells were selected and subsequently gated on IgM-, GFP+ and IgG+. Antigen-specific Ramos cells were double positive for the SARS-CoV-2 S (AF647 and BV421) and ConM Env (AF647 and BV421).
Calcium flux assay: Ramos cells were first gated based on the morphology (FSC-A/SSC-A) and doublets were removed. Live cells were selected and subsequently gated GFP+, Ca2+ influx of B cells is evaluated by the 379/450 nm emission ratio of Indo-1 fluorescence upon UV excitation

Binding assays to cell surface expressed CoV-S :
HEK293 T cells were first gated based on the morphology (FSC-A/SSC-A) and doublets were removed and IgG-PE signal is evaluated in the singlet gate, as representative for binding of the tested monoclonal antibody.

Antibody-dependant cellular phagocytosis Trogocytosis:
ADCT was calculated by the fraction of THP-1 cells that received membrane fragments from the HEK293F cells. To exclude measurement of antibody-independent trogocytosis, cells were gated on stained HEK293F and THP-1 cells in the absence of antibodies. THP-1 and HEK293F cells were first gated based on the morphology (FSC-A/SSC-A) and doublets were removed, THP-1 were selected based on CFSE staining. Frequency of positive ADCT THP-1 is then evaluated based on PKH26 staining. .

Antibody-dependant cellular phagocytosis:
ADCP was calculated by the fraction of THP-1 cells that captured fluorescent beads. THP-1 cells were first gated based on the morphology (FSC-A/SSC-A) and doublets were removed, Frequency of positive ADCP THP-1 is then evaluated based on MFI and frequency of bound beads reflected by postive cells in the FITC chanel.

☒ Tick this box to confirm that a figure exemplifying the gating strategy is provided in the Supplementary Information.
